# PD-1 inhibitor combined with SBRT, GM-CSF, and thymosin alpha-1 in metastatic breast cancer: A case report and literature review

**DOI:** 10.1097/MD.0000000000039271

**Published:** 2024-08-23

**Authors:** Jiamin Yu, Qiang Wang, Lijun Wang, Dan Zong, Xia He

**Affiliations:** a Department of Radiotherapy, The Affiliated Cancer Hospital of Nanjing Medical University and Jiangsu Cancer Hospital and Jiangsu Institute of Cancer Research, Nanjing, China; b Department of Environmental Genomics, Jiangsu Key Laboratory of Cancer Biomarkers, Prevention and Treatment, Collaborative Innovation Center for Cancer Personalized Medicine, Nanjing Medical University, Nanjing, China; c Department of Radiotherapy, Xuzhou Cancer Hospital, Xuzhou, China.

**Keywords:** granulocyte-macrophage colony-stimulating factor (GM-CSF), immunotherapy, metastatic triple-negative breast cancer (mTNBC), refractory solid cancers, stereotactic body radiotherapy (SBRT), thymosin alpha-1, triple-negative breast cancer (TNBC)

## Abstract

**Rationale:**

Triple-negative breast cancer is characterized by a worse prognosis compared with other breast cancer subtypes, especially in the case of pretreated metastatic triple-negative breast cancer (mTNBC). Because of the limited treatment options and suboptimal response rates, there is a pressing need to explore novel treatment protocols.

**Patient concerns:**

A 48-year-old female patient diagnosed with mTNBC who had not responded to multiple lines of therapy (including surgery, chemotherapy, and radiotherapy) but demonstrated significant efficacy and abscopal effects after enrolling in our clinical trial.

**Diagnoses:**

Triple-negative breast cancer with lung metastases.

**Interventions:**

The clinical trial combined stereotactic body radiotherapy, immunotherapy, granulocyte-macrophage colony-stimulating factor, and thymosin alpha-1 to treat previously treated metastatic solid cancers.

**Outcomes:**

This combined treatment regimen implemented in this clinical trial yielded the patient’s notable efficacy, accompanied by abscopal effects. The target lesion and the 3 observed lesions achieved a partial response according to the RECIST v1.1 criteria. reevaluation scans after 2 cycles of immunotherapy indicated a regression rate of −78.97% for the target lesion and −56.73% for the observed lesions. Hematological indexes were stable, and there was no apparent myelosuppression. Also, the tumor marker CA-199 exhibited a downward trend. During the course of treatment, the patient experienced a grade 2 skin reaction, which improved after receiving antiallergic treatment. No further adverse effects were observed.

**Lessons:**

This treatment regimen may offer a promising treatment strategy for patients with mTNBC and other metastatic solid cancers.

## 1. Introduction

Breast cancer is currently the most prevalent malignant tumor in women worldwide.^[1]^ The triple-negative breast cancer (TNBC) subtype is defined by negative estrogen receptor (ER-), negative progesterone receptor (PR-) and negative human epidermal growth factor receptor 2 (HER2-) expression and accounts for 15% to 20% of all breast cancer cases.^[2,3]^ TNBC has a worse prognosis compared with other subtypes.^[4]^ The median overall survival for metastatic TNBC (mTNBC) patients is 18 months, which is significantly lower compared with HR-positive and HER2-enriched patients, in which survival may exceed 5 years.^[5]^ The IMpassion131 and Keynote355 studies demonstrated that PD-(L)1 inhibitors combined with chemotherapy significantly extended progression-free survival (PFS) and overall survival in PD-L1-positive patients, indicating promising results for this treatment strategy. However, monotherapy of pembrolizumab demonstrated a 4.7% response rate in previously treated patients with distant mTNBC, irrespective of PD-L1 status.^[6]^ Thus, effective therapies for pretreated mTNBC patients are urgently needed.

Radiotherapy not only induces DNA damage but also generates immunogenicity and the abscopal effect, which is tumor regression in unirradiated lesions. Previous studies showed that combining immunotherapy with stereotactic body radiotherapy (SBRT) can achieve a synergistic effect.^[7]^ To further stimulate the body’s immune system, new combinations should be explored. Clinical studies demonstrated the safety and efficacy of granulocyte macrophage colony-stimulating factor (GM-CSF) in combination with PD-1 inhibitors and radiotherapy (RT) in treating advanced solid tumors, including breast cancer.^[8,9]^ Thymosin alpha-1 is used to enhance antigen presentation, activate the immune system,^[10]^ and regulate the tumor microenvironment (TME), which may help improve efficiency without increasing toxicity.^[11–13]^

To help broaden the potential population that may benefit from treatment and explore treatments that may enhance the abscopal effect, a multi-center prospective phase II clinical study is conducting to treat patients with advanced recurrent metastatic solid tumors using a combination of SBRT, immunotherapy, GM-CSF and thymosin alpha-1. This study was approved by the Ethics Committee of Jiangsu Cancer Hospital and Xuzhou Cancer Hospital, and registered at Chinese Clinical Trial Registry (ChiCTR2200062706). Here we report a patient who was diagnosed with mTNBC who had not responded to multiple lines of therapy; the patient was enrolled in this clinical trial, and the combination treatment demonstrated significant efficacy and an abscopal effect.

## 2. Case presentation

**Figure 1.**
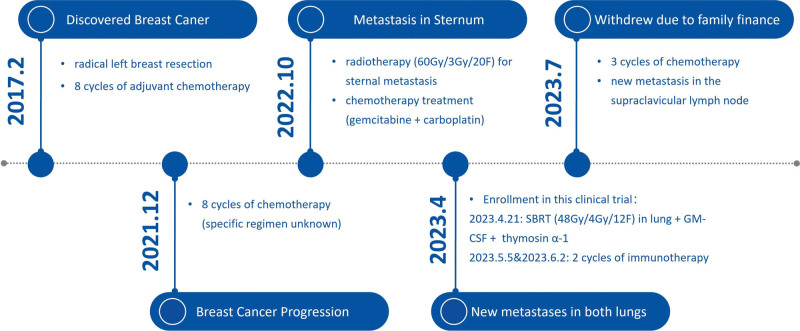
The timeline of disease progression in the patient.

A 48-year-old female patient was admitted to Xinyi Hospital of Traditional Chinese Medicine in February 2017 after a mass was discovered in the left upper chest wall. She underwent a radical left breast resection, and the postoperative pathology revealed breast cancer with ER-, PR-, and HER2- status and 80% Ki-67-positive nuclei. After the surgery, the patient underwent 8 cycles of adjuvant chemotherapy; the specific regimen is unknown. Adjuvant radiation therapy was not administered. At the end of 2021, she began to experience pain in the left chest wall, and the follow-up examination revealed that the tumor had advanced. The patient underwent 8 cycles of chemotherapy at the local hospital. The last chemotherapy treatment was on October 2, 2022. The patient was then admitted to Xuzhou Cancer Hospital and underwent a contrast-enhanced CT on October 25, 2022. The results indicated the presence of a metastasis in the sternal stem in the left upper chest wall. After ruling out contraindications, the patient received radiotherapy (60 Gy/3 Gy/20 F) for sternal metastases of the left upper chest wall on October 25, 2022. The patient underwent chemotherapy treatment (gemcitabine + carboplatin) in this cycle on March 9, 2023. After reevaluation, multiple nodules were discovered in both lungs, which were considered new metastases. For breast cancer patients who have progressed despite multiple lines of therapy, there is currently no treatment option. Therefore, this patient was enrolled in our clinical trial. The timeline of disease progression in the patient is shown in Figure 1.

**Figure 2.**
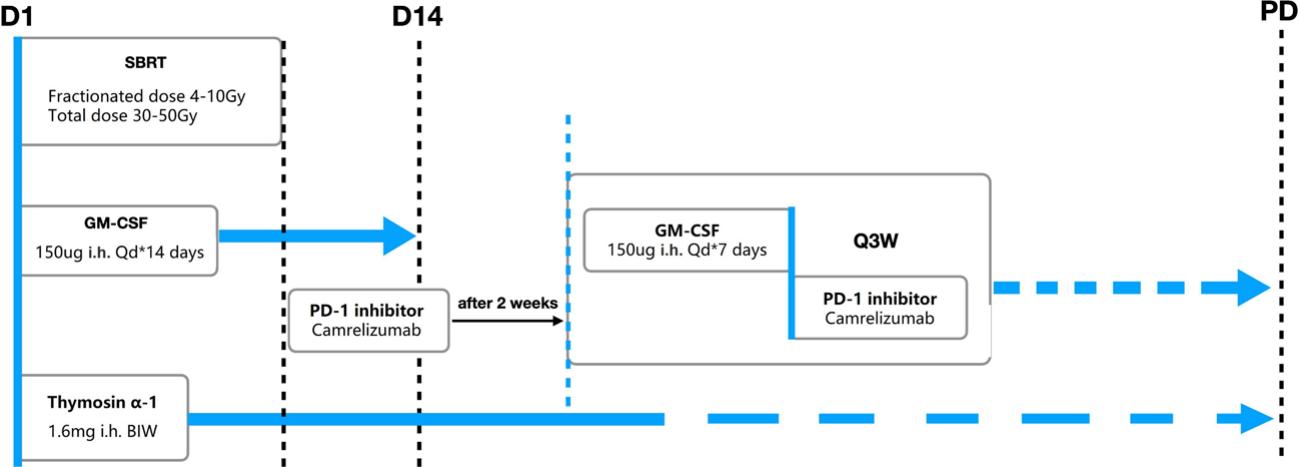
The treatment schedule.

At the time of enrollment, there were 4 measurable metastases located in the lungs and had a history of hepatic sclerosis. The lesion in the right lower lung was treated with SBRT (48 Gy/4 Gy/12 F) on April 21, 2023. GM-CSF at 150 µg was subcutaneously administered daily for 14 days starting from the first day of radiotherapy. Thymosin alpha-1 (1.6 mg, subcutaneous injection, twice weekly) was started until disease progression or the study endpoint. On May 5, 2023, the patient received the first cycle of immunotherapy using Camrelizumab. GM-CSF was then given (150 µg subcutaneously twice a week for 7 days or until white blood cell count increased to 40,000/µL) 1 week prior to the PD-1 inhibitor treatment until disease progression or the study endpoint. The treatment schedule is shown in Figure 2.

**Figure 3.**
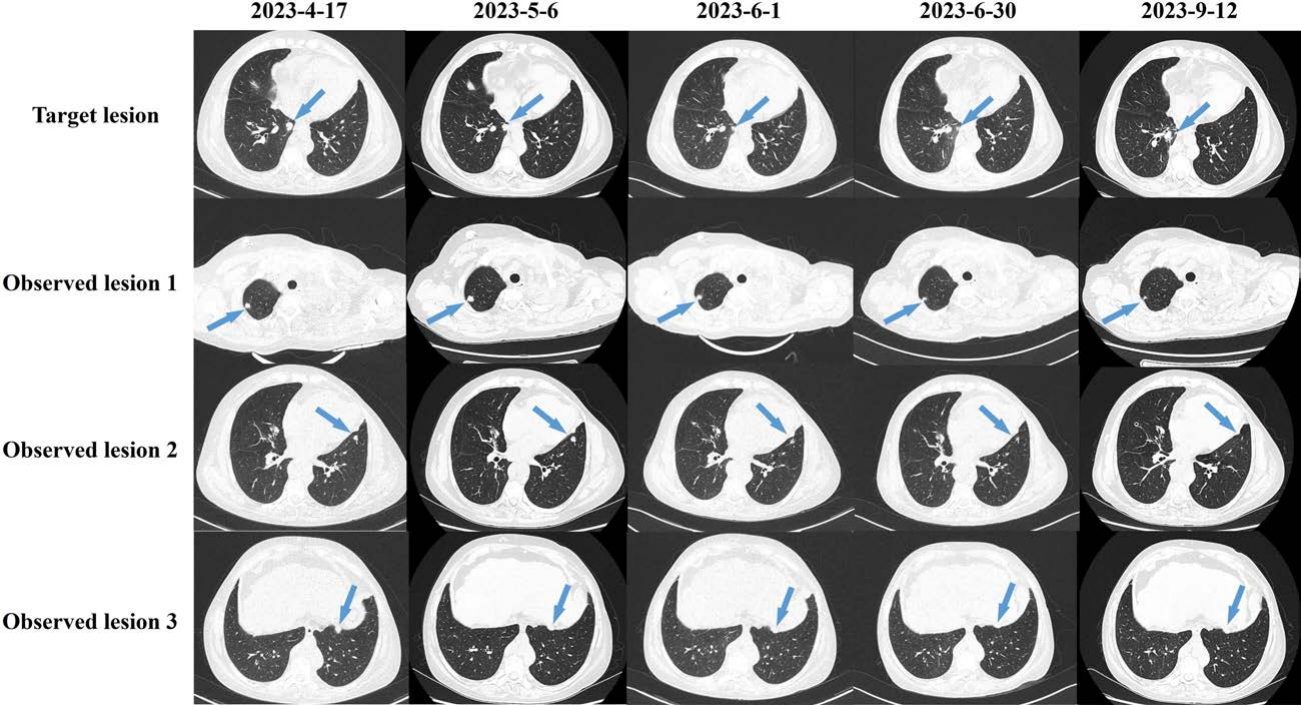
CT scans throughout the treatment. The target lesion is the site that was irradiated, and the observed lesions are the metastases without irradiation. The blue arrows indicate the lesion location.

On May 6, 2023, the patient finished SBRT and the first cycle of immunotherapy, she underwent reevaluation with a contrast-enhanced CT scan. The regression rate of the target lesion was −26.17%, and the regression rate of the observed lesions was −22.64%. After 1 cycle of immunotherapy, on June 1, 2023, the regression rate of the target lesion was −53.75%, and the regression rate of the observed lesions was −37.40%. Both the target lesion and observed lesions achieved partial response (PR) following the RECIST v1.1 criteria. On June 30, 2023, reevaluation scans after 2 cycles of immunotherapy indicated a regression rate of −78.97% for the target lesion and −56.73% for the observed lesions. Both the target lesion and the observed lesions showed increased efficacy (Fig. 3). Over the course of treatment, the patient experienced a grade 2 skin reaction, which improved after receiving anti-allergic treatment. No further adverse effects were observed. After 2 cycles of immunotherapy, the patient withdrew from the clinical trial because of family financial reasons.

The patient received 2 cycles of chemotherapy with vinorelbine (30 mg, day 1, day 8) on July 5 and August 4, 2023. During the treatment, the patient experienced chest tightness, palpitations, bone marrow suppression, and thrombocytopenia, so treatment was paused to relieve the symptoms. On August 21, 2023, the chemotherapy regimen was changed to Halaven (1.8 mg, day 1, day 8) and treatment was continued. As of September 12, 2023, the target lesion could not be detected on CT scans. The observed lesions 1 and 2 have remained almost unchanged since the last screening, while the observed lesion 3 showed a slight progression (10.98%). A new metastasis was observed in the supraclavicular lymph node. The patient had a PFS of 144 days.

**Figure 4.**
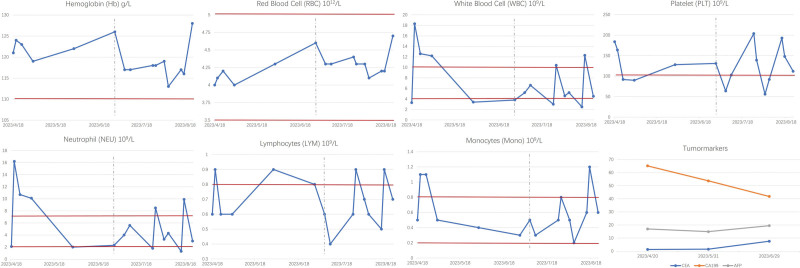
Changes in hematological indexes. The changes in the following hematological indexes during the treatment are shown: hemoglobin, red blood cells, white blood cells, neutrophils, lymphocytes, monocytes, and tumor markers. Red lines represent the lower and upper limits of normal, the gray dashed line represents the time of the last examination in the clinical trial, and the blue lines represent changes in patient indexes.

Blood tests were conducted before each treatment (Fig. 4). Before withdrawal of the clinical trial, the hematological indexes were within normal ranges or slightly lower than the lower limit of normal, and there was no apparent myelosuppression, indicating good tolerance to the treatment. The tumor marker CA-199 exhibited a downward trend. After the patient received chemotherapy, the white blood cell and platelet counts decreased significantly, and drugs for leucopenia and thrombocytopenia were needed before chemotherapy. During the second cycle of chemotherapy, the patient experienced chest tightness and palpitations. Platelet count decreased to 5.6 × 10^10^/L, and there was an increase in total bilirubin, direct bilirubin, and indirect bilirubin levels (26.5 µmol/L, 7.5 µmol/L, 19 µmol/L), indicating hepatocellular damage. As a result, the second half of the chemotherapy was terminated. The safety benefits of the combined treatment are evident.

## 3. Discussion

Several studies have been conducted to explore effective treatments for mTNBC. Currently, there are no widely recognized and effective treatments for mTNBC that failed multiple lines of therapy. Notably, the treatment combining SBRT, immunotherapy, GM-CSF and thymosin alpha-1 exhibited great efficacy in the current case and induced abscopal effects. In a previous study, the combination of radiotherapy and pembrolizumab was well-tolerated and showed promising effectiveness for TNBC in patients who had received multiple lines of therapy (overall response rate, ORR = 17.6%, median progression-free survival, mPFS = 2 months).^[14]^ In our case, the efficacy was superior to these previous results.

Immunotherapy has transformed the treatment landscape for a variety of solid tumors and demonstrated better results than traditional chemotherapy with regard to therapeutic safety.^[15,16]^ However, the response rate of immune checkpoint therapy in TNBC is low, and the effectiveness of PD-1/PD-L1 inhibitors in combination with chemotherapy is limited, as it only receives a level III recommendation in the guideline. A previous study on pembrolizumab monotherapy demonstrated a 4.7% response rate in previously treated patients with distant metastatic TNBC, irrespective of PD-L1 status.^[6]^ In KEYNOTE 119,^[17]^ pembrolizumab monotherapy did not significantly improve overall survival in pretreated mTNBC patients compared with chemotherapy. The results of the IMpassion130 study indicated that patients diagnosed with mTNBC, with PD-L1-negative status or who experienced progressive disease (PD) while receiving immune checkpoint inhibitor therapy, may not have effective treatment options.^[18]^ Therefore, pretreated mTNBC patients may be a suitable population for a combined approach involving immunotherapy and RT.

SBRT generates immunogenicity and abscopal effects by regulating the release of pro-inflammatory mediators, reshaping the tumor microenvironment, and increasing immune stimulation.^[7,16,19]^ However, the underlying mechanism by which radiation therapy affects immune cells and induces distant tumor regression remains inconsistent. Several clinical trials have been conducted worldwide to investigate the best approach for combining radiotherapy with immunotherapy, especially focusing on radiation doses and fractions, aiming to enhance the occurrence of the abscopal effect and broaden the population that can benefit from this treatment.

The occurrence of meaningful abscopal effects is rare. Dose and fractions, tumor burden, number of targets, and site of targets may influence the occurrence of abscopal effects. The significance of fractionated doses of radiotherapy was demonstrated in a study that revealed activation of immune response–related genes, radiation-induced damage-associated molecular pattern molecules, and inflammatory cytokines after treatment with radiation in the range of 6 to 10 Gy.^[20]^ This activation further stimulates anti-tumor immune responses. Conversely, higher doses, such as ≥ 15 Gy, increase the proportion of regulatory T (Treg) cells in the spleen, which have the ability to suppress tumor-specific immunity.^[21]^ In the current case, SBRT was administered to the lung metastasis with a single fraction dose of 4 Gy. While this dose was lower than those reported, it still induced abscopal effects. The use of GM-CSF and thymosin alpha-1 may contribute to these effects by modifying the tumor microenvironment. These findings potentially pave the way for a novel treatment approach for heavily treated mTNBC.

GM-CSF is a cytokine that stimulates the production of neutrophils, monocytes, macrophages, and dendritic cells, influencing host immune surveillance by regulating the function of innate immune cells and serving as a bridge to activate adaptive immune responses.^[8]^ Clinical studies have demonstrated the safety and efficacy of GM-CSF in combination with PD-1 inhibitors in treating advanced solid tumors, including breast cancer.^[8,9]^ Thymosin alpha-1 is a traditional immune-enhancing medication that enhances antigen presentation, activates the body’s innate and adaptive immune systems,^[10]^ and increases the infiltration of tumors by CD4 + T and CD8 + T cells^[22]^ to regulate the tumor microenvironment.^[13]^ It may be possible to broaden the scope of patients who could benefit from this combination therapy regimen without escalating toxicity and side effects.^[11–13]^

### 3.1. Limitations

This case report is only representative of this patient’s situation, and the effectiveness of this treatment regimen requires more patients to enroll and further analysis.

## 4. Conclusion

The combination of radiotherapy and immunotherapy is a promising treatment approach for cancer patients, and optimizing the dosage when combining these 2 treatments is currently a hot topic in research. This case report demonstrates the great efficacy of combining immunotherapy with radiotherapy, GM-CSF, and thymosin alpha-1 in treating a breast cancer patient who did not respond to multiple lines of advanced therapy. Our observations also suggest the potential of this treatment in inducing abscopal effects, offering a new treatment approach for patients with advanced solid tumors.

## Acknowledgments

We thank Gabrielle White Wolf, PhD, from Liwen Bianji (Edanz) (www.liwenbianji.cn) for editing the English text of a draft of this manuscript. The authors thank to the patient and her family for their support of this study.

## Author contributions

**Conceptualization:** Lijun Wang, Xia He.

**Data curation:** Jiamin Yu.

**Formal analysis:** Jiamin Yu.

**Funding acquisition:** Xia He.

**Investigation:** Lijun Wang.

**Methodology:** Lijun Wang, Dan Zong.

**Project administration:** Lijun Wang, Xia He, Qiang Wang.

**Resources:** Qiang Wang.

**Software:** Jiamin Yu.

**Supervision:** Dan Zong, Xia He.

**Validation:** Qiang Wang.

**Visualization:** Jiamin Yu.

**Writing – original draft:** Jiamin Yu.

**Writing – review & editing:** Lijun Wang, Dan Zong.
